# Contribution of the genetic background to the immune response of broilers vaccinated or challenged with LPAI H9N2

**DOI:** 10.1186/1753-6561-5-S4-S5

**Published:** 2011-06-03

**Authors:** Lonneke Vervelde, Eveline de Geus, Christine Jansen, Dan E Heller

**Affiliations:** 1 Dept. Infectious Diseases and Immunology, Faculty Veterinary Medicine, University Utrecht, Yalelaan 1, 3584 CL Utrecht, The Netherlands; 2 Dept of Animal Sciences, Faculty of Agricultural, Food and Environmental Quality, The Hebrew University of Jerusalem, P.O.Box 12, Rehovot 76100, Israel

## Abstract

**Background:**

The knowledge on the immune responses to LPAI is limited. The purpose of this study was to investigate the immune responses of two divergently selected lines of broilers, a line responding with high antibody response to antigens (HH), and a line responding with low antibody titers (LL) to antigen.

**Methods:**

Day old chicks from each line were divided in two groups, one vaccinated with inactivated H9N2 vaccine and one non-vaccinated. At 21 days of age all the chicks were challenged with field isolate of H9N2, 1X10^6.5^ ELD_50_ per chick by drops to the eye, nose and beak. Twenty four hours and 14 days post challenge (PC), the chickens were weighed blood spleen and lungs were taken and leukocytes were isolated. The leukocytes were stained with monoclonal antibodies for surface markers and analyzed by flow cytometry. We used Elispot assay to identify the number of antibody producing cells in each of the organs. mRNA was extracted using TRIsol reagent in order to assess the cytokine production level by qRT-PCR using the SYBR green methods.

**Results:**

Our results showed that LL-vaccinated group gained more weight than any of the other group. Using IDEXX kit, no antibody titers could be identified in vaccinated chicks 21 days post vaccination while 14 days PC vaccinated HH chickens demonstrated the highest average antibody titers. LL vaccinated chickens demonstrated higher average antibody titer than non-vaccinated LL. Using the Elispot assay no difference were found between the groups either cells producing IgA, IgM or IgY beside of a high number of IgY producing cells in the lungs of vaccinated HH birds.

**Conclusions:**

Further data on leukocytes subpopulations using flow cytometry, cytokines production (IFNγ, IL-6, IL-18, IL-2 and IL-4) isotype specific antibody responses and number and functionality of NK cells are in process.

## Background

Influenza viruses of type A infect humans, horses, swine other mammals and a wide variety of domesticated and wild birds. The reservoir of the virus is considered to be in wild waterfowl. Infection of poultry with AI viruses cause a wide range of clinical signs including mild and severe respiratory disease, producing losses and sometimes severe disease with high morbidity and mortality. AI viruses are typically characterized as either being Low Pathogenic Avian Influenza (LPAI), or High pathogenic AI (HPAI) viruses. Numerous vaccines against avian influenza (AI) have been developed and shown to be efficacious, but the number of AIV outbreaks in commercial poultry is reduced but not eradicated. With a greater understanding of the host immune response to the AI infection and vaccination, better control strategies can be developed.

Differences in pathogenicity between species have been observed in galliforme birds in experimental studies with LPAI and HPAI viruses [1]. Differences are also apparent when comparing the immune responses, primarily antibody titers, of different species of AI virus infections.

Our knowledge of avian cellular immunology has expanded rapidly in the last decade. It is well accepted that the cellular immune response is important in the defense against many viral infections. However, very little is known about the importance of cellular immunity against AI virus.

The objective of this work was to study the interaction of inactivated LPAI virus vaccine and the challenge with virulent LPAI on the immune system of chickens.

An important part of this project was to analyze the influence of genetic factors on chicken immune responses against LPAI using two divergently selected broiler lines.

These genetically distinct chicken lines identified as high (HH) or low (LL) responders in respect to antibody responses [2,3].

## Methods

Chicks of the HH and LL lines hatched in the Utrecht University facilities. At day old chicks each line was divide into two, half vaccinated and Half non-vaccinated.

Vaccination was performed injecting subcutaneously 0.5 ml of inactivated A/IL/H9N2/125 vaccine (log_10_ 3.8 EID_50_). Non- vaccinated chicks were subcutaneously injected with 0.5 ml PBS. At 21 days of age all the chicks were weighed and bled, and challenged with 0.1 ml = log_10_ 6.5 EID_50_ H9N2/ chick by nose and eye-drops. The serum was used for antibody determination by IDEXX kit.

At 22 days of age (One day post challenge (dpc) for early response), 4HH non-vaccinated and 4HH vaccinated, 5LL non-vaccinated and 5LL vaccinated were taken, blood spleen and lungs were sampled. At 35days old (14 dpc for late response) 2 HH non-vaccinated and 4 HH vaccinated, 5 LL non-vaccinated and 5 LL vaccinated chickens were taken, and blood, spleen and lungs were harvested.

### Leukocytes isolation

Blood was taken with anticoagulant (Heparine), diluted by equal volume of PBS at room temperature (RT), and was over layered on Ficoll (GE Healthcare,Uppsala) and centrifuged 20 min at 2200 rpm. The interphase containing the leukocytes was harvested washed twice and finally re-suspended in 5 ml and counted using trypan blue and haemocytometer.

Spleen leukocytes were isolated by removing the capsule and pressing the spleens through cell strainer (BD Falcon, Bedford, MA.). The cells were overlayered on Ficoll (GE Healthcare,Uppsala) and treated as the blood lymphocytes. The cells harvested from the interphase were washed twice (1,300 rpm for 5 min), re-suspended in 20 ml and counted.

Lung leukocytes were isolated by cutting the lungs into small pieces in 6 wells plated. To each well 2ml of collagenase/DNAse solution was added. The plates were incubated for 30 min at 37^0^C with 5% CO_2_. The lung tissue was pressed through 70 mM cell strainer (BD Falcon, Bedford, MA.). The cells were washed once (1300 rpm for 5 min.), re-suspended in 5 ml PBS and overlayered on Ficoll, and the interphase collected. The cells were washed twice (1,300 rpm for 5 min), re-suspended in 5 ml and counted.

## Results

As it can be seen from table 1, LL vaccinated chicks gained more weight than the other 3 experimental groups. By the end of the experiment at 35 days of age LL vaccinated chickens were heavier than the other 3 groups (Table 2.)

**Table 1:** Mean body weight (BW) of the 4 experimental groups of chick at 21 days of age (g)

	HH Non-vaccinated	HH Vaccinated	LL Non-vaccinated	LL Vaccinated
BW	368.7	370.3	381.5	454.8

**Table 2:** Mean body weight (BW) of the 4 experimental groups of chicken at 35 days of age (g)

	HH Non-vaccinated	HH Vaccinated	LL Non-vaccinated	LL Vaccinated
BW	1152.5	1094.0	1110.8	1351.0

**Figure 1:**
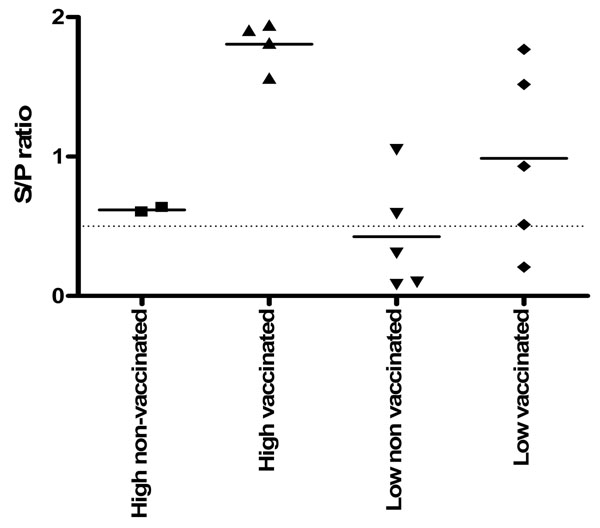
Serum antibody titers of 35 days old chickens 14 days post challenge with AI H9N2

**Figure 2:**
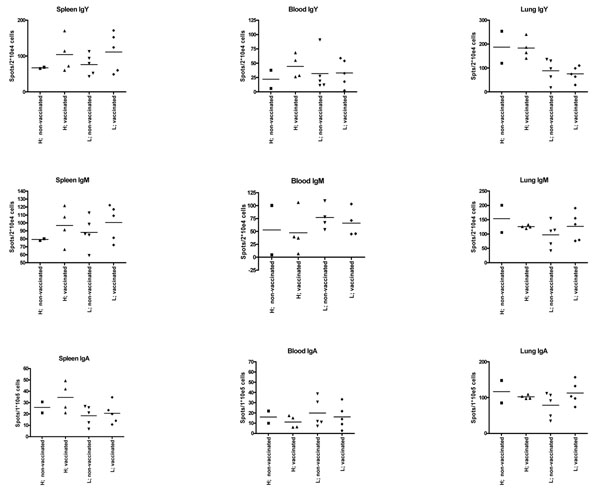
**Plasma cell frequencies in one of the 3 isotypes, IgM, IgY or IgA in lung, spleen and blood at 14 days post challenge**. Elispots analysis of the 4 groups, when lymphocytes isolated from spleens, blood and lungs 14 dpc exhibited no difference between groups, organs or isotype. Only in the lungs high number of IgY producing cell were found in the two HH groups while in the LL groups low IgY producing cells could be detected.

No H9N2 specific serum antibody titers could be detected at 21 days of age. At 35 days of age (14 dpc), no antibody titers could be detected in non-vaccinated chickens (Fig.1.), while in the HH vaccinated chickens high antibody titers could be detected. In the LL vaccinated group 2 chickens responded with high titer, 2 responded with low and one gave average titer. Using the Elispot assay no difference were found between the groups either cells producing IgA, IgM or IgY beside of a high number of IgY producing cells in the lungs of vaccinated HH birds (Fig.2.)

## Discussion

Chicks vaccinated at one day of age with an inactivated vaccine did not responded with a measurable titer of antibodies at 21 days of age. A challenge with a field strain of H9N2 resulted in a specific antibody titers in vaccinated chickens only. HH vaccinated and challenged group responded with High antibody titers but also with a high number of IgY positive cells in the lungs

## Competing interests

The authors declare that they have no competing interests.
